# AGE-RELATED CHANGES TO MACROPHAGES AFFECT FRACTURE HEALING

**DOI:** 10.1093/geroni/igz038.321

**Published:** 2019-11-08

**Authors:** Daniel Clark, Theodore Miclau, Mary Nakamura, Ralph Marcucio

**Affiliations:** University of California San Francisco, San Francisco, California, United States

## Abstract

Fracture healing follows a strict temporal sequence characterized by an initial inflammatory phase. Perturbation of the inflammatory phase may be responsible for the poorer fracture healing outcomes in older adults. Herein, we examine age-related changes to the macrophage during fracture healing. Macrophages regulate inflammation through pro-inflammatory (M1) and anti-inflammatory (M2) phenotypes. Anti-inflammatory activity is promoted via activation of triggering receptor expressed on myeloid cells 2 (TREM2). Tibia fractures were made in old (24 months) and young (3 months) mice. Immune cells from the fracture callus were analyzed via RNAseq and FACS, and fracture healing was evaluated histologically. Old mice demonstrated significantly delayed fracture healing compared to young (p<0.05). The quantity of infiltrating macrophages into the fracture callus was similar in young and old mice. However, 1222 genes were significantly differentially regulated (FDR<0.1) in callus macrophages from old mice compared to young, and old macrophages demonstrated a more pro-inflammatory phenotype. TREM2 expression was increased in macrophages after fracture in both groups but was significantly less in old mice compared to young via RNAseq and FACS (FDR<0.1, p<0.05). TREM2-/- mice demonstrated increased pro-inflammatory cytokine expression within the callus with resulting significant delays in fracture healing compared to age-matched controls (p<0.05). Inhibition of macrophage infiltration into the fracture callus significantly improved fracture healing in old mice compared to age-matched controls. Age-related changes to macrophages, including increased pro-inflammatory cytokine expression and dysregulated TREM2 expression, may explain fracture healing deficits observed in older adults. Therapeutically targeting macrophages may improve management of fractures in older adults.

